# The human rs1050286 polymorphism alters LOX‐1 expression through modifying miR‐24 binding

**DOI:** 10.1111/jcmm.12716

**Published:** 2015-11-06

**Authors:** Elena Morini, Barbara Rizzacasa, Sabina Pucci, Chiara Polidoro, Fabrizio Ferrè, Daniela Caporossi, Manuela Helmer Citterich, Giuseppe Novelli, Francesca Amati

**Affiliations:** ^1^ Department of Movement, Human and Health SciencesForo Italico UniversityRomeItaly; ^2^ Department of Biomedicine and PreventionUniversity of Rome Tor VergataRomaItaly; ^3^ Department of BiologyUniversity of Rome Tor VergataRomaItaly

**Keywords:** Hsa‐mir‐24, *OLR*1 gene, acute myocardial infarction, Atherosclerosis, alternative splicing

## Abstract

The up‐regulation of lectin‐like oxidized low‐density lipoprotein receptor‐1 (LOX‐1), encoded by the *OLR1* gene, plays a fundamental role in the pathogenesis of atherosclerosis. Moreover, *OLR1* polymorphisms were associated with increased susceptibility to acute myocardial infarction (AMI) and coronary artery diseases (CAD). In these pathologies, the identification of therapeutic approaches that can inhibit or reduce LOX‐1 overexpression is crucial. Predictive analysis showed a putative hsa‐miR‐24 binding site in the 3′UTR of *OLR1*, ‘naturally’ mutated by the presence of the rs1050286 single nucleotide polymorphism (SNP). Luciferase assays revealed that miR‐24 targets *OLR*1 3′UTR‐G, but not 3′UTR‐A (*P* < 0.0005). The functional relevance of miR‐24 in regulating the expression of *OLR1* was established by overexpressing miR‐24 in human cell lines heterozygous (A/G, HeLa) and homozygous (A/A, HepG2) for rs1050286 SNP. Accordingly, HeLa (A/G), but not HepG2 (A/A), showed a significant down‐regulation of *OLR1* both at RNA and protein level. Our results indicate that rs1050286 SNP significantly affects miR‐24 binding affinity to the 3′UTR of *OLR1*, causing a more efficient post‐transcriptional gene repression in the presence of the G allele. On this basis, we considered that *OLR1* rs1050286 SNP may contribute to modify *OLR1* susceptibility to AMI and CAD, so ORL1 SNPs screening could help to stratify patients risk.

## Introduction

Atherosclerosis is characterized by the formation of plaques on the inner walls of arteries that threatens to become the leading cause of death worldwide *via* its sequelae of acute myocardial infarction (AMI) and stroke. Many studies show that oxidized low‐density lipoproteins (ox‐LDL) play a key role in atherogenesis. Indeed, sub‐endothelial retention of ox‐LDL is considered the initial event of atherogenesis, followed by the infiltration and activation of inflammatory cells circulating in the blood 1. Lectin‐like oxidized low‐density lipoprotein receptor‐1 (LOX‐1) is the main ox‐LDL endothelial scavenger receptor (SR) 2 and the ox‐LDL/LOX‐1 interaction contributes to the triggering of endothelial dysfunction characteristic of atherosclerosis development 1. Moreover, single nucleotide polymorphisms (SNPs) in the *OLR1* gene, encoding LOX‐1, have been associated with the risk of developing AMI 3, 4.

*OLR1* (NM_002543) is subjected to alternative splicing generating two isoforms: Loxin (NM_001172633) 5 and OLR1D4 (NM_001172632). OLR1D4 lacks exon 4, so the putative encoded protein is shorter with a distinct C‐terminus. No other data are available for this isoform. On the contrary, Loxin, the splice isoform lacking exon 5, is well known and characterized 5, 6. It encodes for a truncated receptor (LOXIN) lacking two‐thirds of LOX‐1 functional lectin‐like binding domain. LOXIN forms heterodimers with LOX‐1 reducing the cells’ ability to bind ox‐LDL; when LOXIN is co‐expressed with LOX‐1, it is able to rescue cells from LOX‐1‐induced apoptosis in a dose‐dependent manner 6. Moreover, *in vivo* studies on animal models indicate that LOXIN expression induces a significant decrease in plaque coverage within the common carotid artery 7.

MicroRNAs (miRNAs) are small single‐stranded non‐coding RNAs of 18–23 nucleotides, active in the regulation of gene expression both at transcriptional and post‐transcriptional levels. MicroRNAs, in fact, can block mRNA translation through a partial bond with complementary mRNA targets or determine a complete degradation when pairing is perfectly complementary 8.

Previous studies have described interactions among *OLR1* and different miRNAs; within them, particularly interesting are hsa‐let7‐g 9 and hsa‐miR‐21 10 that are expressed mainly in endothelial cells and are associated with the apoptosis regulation.

To identify new miRNAs modulating the expression of *OLR1* and its splice isoforms, we performed an *in silico* analysis searching for miRNA putative binding sites using popular miRNA target prediction algorithms (TargetScan and miRanda). Among the 89 putative miRNA binding sites identified, we functionally analysed and characterized the putative binding site for hsa‐miR‐24. In fact, the miR‐24 binding site, located in the 3′UTR of *OLR1*, contains a known SNP, rs1050286. Dual luciferase reporter assays showed that miR‐24 inhibited the expression of *OLR1* by binding to the 3′UTR and that the inhibitory role of miR‐24 was impacted by rs1050286 SNP.

In addition, the functional relevance of miR‐24 in regulating physiologically *OLR1* expression was established by miR‐24 overexpression studies in human cell lines heterozygous (A/G; *i.e*. HeLa) and homozygous (A/A; *i.e*. HepG2) for rs1050286 SNP. As expected, HeLa cells (A/G) showed a significant down‐regulation of *OLR1* expression both at RNA and at protein level compared to HepG2 (A/A); moreover, the overexpression of miR‐24 in HeLa cells resulted in a decrement in cell proliferation rate.

Our results demonstrate that *OLR1* is a new target of miR‐24 and that a genetic SNP (rs1050286) may disrupt miR‐24 binding site modulating *OLR1* expression level. On this basis, we postulated that *OLR1* rs1050286 SNP might contribute to modify *OLR1* susceptibility to AMI and CAD.

Even if these data may be validated by additional studies, they suggest that *OLR1* rs1050286 SNP screening could help to stratify patient risk.

## Materials and methods

### miRNA putative binding sites *in silico* analysis

*In silico* analysis were performed on the coding sequence and 3′UTR of *OLR1* gene using TargetScan v6.0 (http://www.targetscan.org/) and miRanda (http://www.microrna.org/) software. All algorithms were run with default parameters. TargetScan predictions were evaluated on genomic multiple alignments of human, mouse, rat, cow, dog and chicken, imposing conservation in at least five species. The TargetScan context component of the score was ignored for the predictions in the coding sequence.

### Luciferase reporter assays

The 3′UTR of *OLR1* containing the putative miR‐24 binding site (G) and the ‘naturally’ mutated site (A) were amplified from genomic GM06991 and GM07053B clones, respectively, and the PCR products were XhoI/NotI digested and subcloned in psiCHECK‐2 vector immediately downstream of the *Renilla Luciferase* gene. The *Firefly Luciferase* gene reporter was used as control for transfection efficiency. pEFDEST51 premiR‐24‐2 vector was used to overexpress miR‐24 11. About 2 × 10^6^ cells have been plated in a 60‐mm Petri dish and transfected with a total of 5 μg of DNA (for the cotransfection, we used 2.5 μg of each plasmid) using *Lipofectamine 2000* (Invitrogen, Waltham, MA, USA). After 24 hrs, a luciferase assay was conducted using Dual Glo^®^ Luciferase Assay (Promega Fitchburg, WI, USA) according to the manufacturer's instructions.

### OLR1 SNP rs1050286 genotyping

Genomic DNA was extracted following the Flexigene Kit protocol (Qiagen, Hilden, Germany). A TaqMan^®^ Genotyping Assay protocol (C_7433809_30; Life Technologies, Waltham, MA, USA) was used to analyse rs1050286 SNP in HeLa and HepG2 cell lines.

### Cell culture and miRNA overexpression

HeLa and HepG2 cell lines (ATCC) were cultured in complete medium DMEM supplemented with 10% Fetal Bovine Serum (FBS), 1× L‐glutamine, 1× Fungizone at 37°C and 5% CO_2_. Cells were seeded in triplicate at a 25,000 cell/cm^2^ density and grown in complete culture medium. Both cell lines were transiently transfected by using Calcium Phosphate Transfection Kit (Invitrogen) with 5 μg of pEFDEST51 premiR‐24‐2 vector and pEGFPN‐1 as control. Cells were harvested 48 and 72 hrs after transfection and suspended in 1 ml of Trizol (Ambion Foster City, CA, USA) (until RNA extraction) for qRT‐PCR assay and fixed in buffered formalin for immunocytochemistry.

### Quantitative real time PCR

Total RNA from transfected cells was treated with DNAse (2 U/μl; Ambion) and then retrotranscribed by using ‘High Capacity cDNA Reverse Transcription Kit’ (Applied Biosystems, Waltham, MA, USA). A qRT‐PCR (SYBR Green assay Applied Biosystems) assay was performed with different primers pairs, designed using the software Primer Blast, specific for each *OLR1* isoforms: OLR1 (F: *5′‐GCACAGCTGATCTGGACTTCAT‐3′*, R: *5′‐CCCCATCCAGAATGGAAAACT‐3′*), LOXIN (F: *5′‐AAAAGAGCCAAGAGAAGTGCTTGT‐3′*, R: *5′‐TCTAAATCAGATCAGCTGTGC‐3′*) and OLR1D4 (F: *5′‐TTGTTCAGGACTTCATCCAGC‐3′*, R: *5′‐TCGGACTCTAAATAAGTGGGG‐3′*). RPL37A and β‐actin genes were used for data normalization.

### Western Blot analysis

Standard protein extraction was performed with RIPA lysis buffer. Denatured protein extracts (35 μg) were loaded on a 10% SDS‐PAGE. Proteins were transferred to a polyvinylidene difluoride (PVDF) membrane (Hybond‐P; Amersham‐Pharmacia Biotech, GE Healthcare Life Sciences, Amersham Place, Little Chalfont, Buckinghamshire, UK). Membranes were stained with Ponceau S dye, to check for equal loading and homogeneous transfer and incubated for 1 hr at RT with 3% skim milk (Difco Lab., Detroit, MI, USA) and 0.5% Tween 20 (USB, Cleveland, OH, USA). The membrane was incubated with anti‐LOX‐1 rabbit policlonal (Abcam, Cambridge, UK) and anti‐β‐actin mouse IgG1 monoclonal antibody (Sigma‐Aldrich, Saint Louis, MO, USA). Horseradish peroxidase‐conjugated antibodies were used as secondary antibodies. Signals were detected by the SuperSignal‐detection method (Thermo Scientific, Waltham, MA, USA) and quantified by densitometry (GeneGnome; SynGene, Bangalore, Karnataka, India) after normalization for β‐actin gene product. All the experiments were repeated three times and gave similar results.

### Immunocytochemistry

An anti‐LOX‐1 antibody (R&D, Minneapolis, MN, USA) was used to evaluate LOX‐1 expression. To assess the background staining, a negative control was carried out without addition of primary antibody. Secondary antibody (Biotinylated goat anti‐rabbit IgG) and following reagents (HRP‐conjugated streptavidin) were added. After washing, slides were incubated with diaminobenzidine and counterstained with haematoxylin.

### Proliferation assay

Transfected cells, seeded in triplicate, at a density of 6 × 10^3^ cells/cm^2^, were counted after 24, 48, 72 and 96 hrs. Trypan blue staining was performed to evaluate cell death percentage. The cell suspension was transferred to a haemocytometer for cell counting.

### Statistical analysis

Each analysis was performed in triplicate and data are expressed as mean values ± SD. Student's *t*‐test was used to compare two groups. A *P* ≤ 0.05 was considered to be significant.

## Results

An *in silico* analysis using miRanda and TargetScan programs was conducted on the entire coding sequence and 3′UTR of *OLR1* and its splice isoforms to identify new miRNAs binding sites. A total of 89 miRNA putative binding sites (Table 1) potentially interacting with *OLR1* were identified. To find known SNPs that could modulate miRNA/*OLR1* gene pairing we analysed them in the miR‐SNP database (http://www.bioguo.org/miRNASNP/). Twenty‐three miRNAs presented SNPs in the binding region (Table 1, in bold).

**Table 1:** List of the 89 putative miRNAs identified on coding and 3′UTR sequences of *OLR1*. The start and end position of each seed region are indicated. In bold are shown the 23 miRNAs containing a known SNP in the seed region

miRNAs	OLR1 (NM_002543)			
	Start match	End match	TargetScan Score	miRanda Score
**hsa‐miR‐298**	**35**	**42**	**−0.357**	**145**
**hsa‐miR‐3158‐5p**	***37***	**44**	**−0.373**	**143**
**hsa‐miR‐4274**	**77**	**83**	**−0.23**	**142**
hsa‐miR‐4693‐3p	85	91	−0.283	155
hsa‐miR‐767‐5p	274	280	−0.23	159
hsa‐miR‐3065‐3p	276	282	−0.233	155
**hsa‐miR‐4266**	**309**	**316**	**−0.366**	**142**
**hsa‐miR‐1258**	**311**	**318**	**−0.287**	**153**
hsa‐miR‐3152‐3p	313	320	−0.25	142
**hsa‐miR‐581**	**316**	**323**	**−0.279**	**149**
hsa‐miR‐4464	329	336	−0.237	NA
hsa‐miR‐4299	339	346	−0.361	142
hsa‐miR‐345	403	410	−0.325	155
hsa‐miR‐615‐3p	442	448	−0.265	NA
hsa‐miR‐876‐5p	459	466	−0.257	147
hsa‐miR‐3167	459	466	−0.278	140
hsa‐miR‐4456	486	493	−0.439	140
hsa‐miR‐620	500	507	−0.316	147
hsa‐miR‐1270	500	507	−0.327	NA
hsa‐miR‐432	502	509	−0.4	147
hsa‐miR‐4754	545	552	−0.569	150
hsa‐miR‐194	576	582	−0.246	140
**hsa‐miR‐4642**	**640**	**647**	**−0.336**	**140**
hsa‐miR‐198	675	681	−0.233	NA
**hsa‐miR‐1287**	**687**	**692**	**−0.356**	**147**
hsa‐miR‐3151	786	792	−0.339	150
**hsa‐miR‐3917**	**802**	**808**	**−0.295**	**157**
hsa‐miR‐3192	816	823	−0.344	151
**hsa‐miR‐4268**	**857**	**863**	**−0.233**	**NA**
hsa‐miR‐718	871	877	−0.242	NA
hsa‐miR‐3614‐3p	941	947	−0.256	168
hsa‐miR‐520a‐5p	946	953	−0.308	140
hsa‐miR‐525‐5p	946	953	−0.318	146
hsa‐miR‐96	1012	1019	−0.315	141
hsa‐miR‐1271	1012	1019	−0.336	NA
hsa‐miR‐4674	1025	1031	−0.264	140
hsa‐miR‐378g	1026	1033	−0.384	141
hsa‐miR‐1184	1051	1058	−0.35	NA
**hsa‐miR‐544**	**1066**	**1073**	**−0.244**	**NA**
hsa‐miR‐4755‐3p	1088	1095	−0.446	150
hsa‐miR‐1302	1147	1154	−0.364	146
hsa‐miR‐4298	1147	1154	−0.385	157
**hsa‐miR‐1207‐5p**	**1159**	**1166**	**−0.474**	**172**
hsa‐miR‐4763‐3p	1159	1166	−0.421	156
**hsa‐miR‐498**	**1217**	**1224**	**−0.281**	**NA**
hsa‐miR‐1248	1376	1383	−0.238	NA
hsa‐miR‐4757‐5p	1385	1392	−0.403	151
hsa‐miR‐140‐3p	1455	1461	−0.262	161
hsa‐miR‐4451	1473	1480	−0.382	154
hsa‐miR‐3120‐5p	1489	1496	−0.267	161
hsa‐miR‐3120‐5p	1493	1500	−0.3	161
hsa‐miR‐3120‐5p	1497	1505	−0.312	161
hsa‐miR‐4286	1539	1546	−0.375	140
hsa‐miR‐4674	1545	1552	−0.378	NA
hsa‐miR‐635	1547	1554	−0.433	143
hsa‐miR‐4752	1592	1599	−0.326	143
hsa‐miR‐4795‐5p	1607	1613	−0.234	152
**hsa‐miR‐4650‐3p**	**1644**	**1651**	**−0.379**	**NA**
**hsa‐miR‐3909**	**1651**	**1658**	**−0.364**	**142**
**hsa‐miR‐3611**	**1676**	**1683**	**−0.284**	**NA**
hsa‐miR‐3153	1736	1742	−0.246	NA
hsa‐miR‐1253	1770	1777	−0.272	150
hsa‐miR‐567	1806	1813	−0.242	140
hsa‐miR‐297	1807	1814	−0.286	144
hsa‐miR‐643	1809	1816	−0.23	NA
hsa‐miR‐4658	1831	1838	−0.337	140
**hsa‐miR‐4777‐5p**	**1838**	**1845**	**−0.266**	**NA**
hsa‐miR‐4481	1936	1942	−0.265	146
hsa‐miR‐4745‐5p	1936	1942	−0.265	140
**hsa‐miR‐24**	**1942**	**1964**	**−0.267**	**141**
**hsa‐miR‐516a‐3p**	**1965**	**1971**	**−0.265**	**157**
hsa‐miR‐3944‐5p	1983	1990	−0.503	151
hsa‐miR‐3074‐5p	1998	2004	−0.254	157
**hsa‐miR‐4639‐3p**	**2172**	**2179**	**−0.419**	**141**
hsa‐miR‐3201	2181	2188	−0.319	140
hsa‐miR‐4791	2181	2188	−0.351	154
hsa‐miR‐3134	2184	2191	−0.356	NA
hsa‐miR‐4459	2214	2221	−0.33	144
**hsa‐miR‐4667‐5p**	**2229**	**2236**	**−0.376**	**158**
**hsa‐miR‐4700‐5p**	**2229**	**2236**	**−0.366**	**NA**
**hsa‐miR‐876‐3p**	**2238**	**2245**	**−0.371**	**147**
hsa‐miR‐518a‐5p	2327	2333	−0.237	NA
hsa‐miR‐527	2327	2333	−0.237	NA
hsa‐miR‐4534	2357	2363	−0.253	141
hsa‐miR‐4272	2395	2402	−0.253	142
hsa‐miR‐4506	2434	2440	−0.283	146
hsa‐miR‐4733‐5p	2473	2480	−0.393	161
hsa‐miR‐646	2491	2497	−0.283	143
hsa‐miR‐578	2512	2518	−0.261	NA

**Figure 1:**
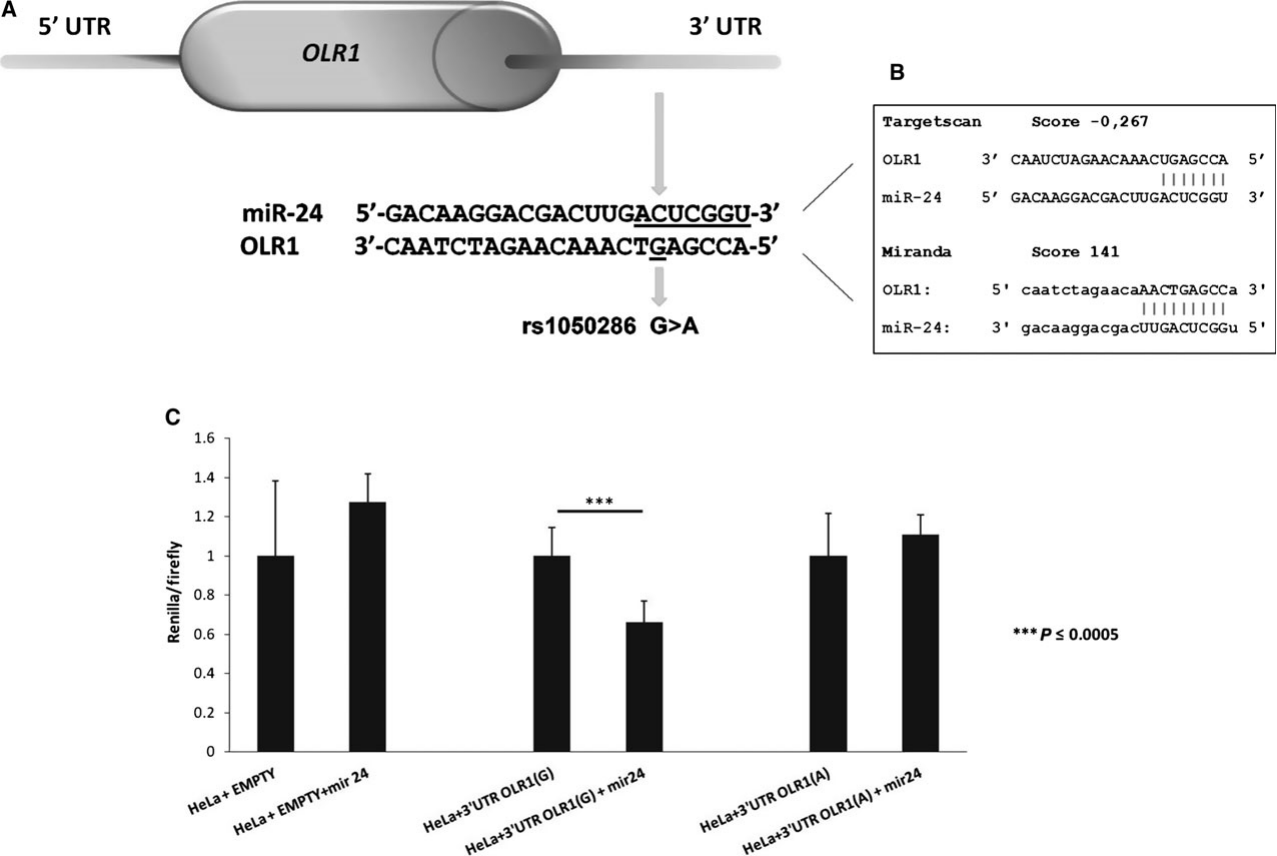
Effect of miR‐24 overexpression on *OLR1* 3′ UTR luciferase activity. (**A**) A graphic representation of miR‐24 binding site on *OLR1* 3′UTR. The miR‐24 seed region is underlined. The position of SNP rs 1050286 is shown. (**B**) Prediction scores of the interaction between *OLR1* and miR‐24 by TargetScan and miRanda software. (**C**) Luciferase assay results. Data were normalized to Firefly luciferase activity and to cells transfected with control vectors (empty psiCHECK‐2 or 3′UTR(G) alone or 3′UTR(A) alone). Error bars represent standard deviation of technical repeat experiments (*n* = 3).

Among them, rs1050286 SNP (G>A), mapping in the 3′UTR of the *OLR1* gene, is located within the seed‐binding region for hsa‐miR‐24 (Fig. 1A and B).

To test the interaction between *OLR1* 3′UTR and miR‐24, we performed an *in vitro* luciferase assay. HeLa cells transfected with a plasmid expressing *OLR1* 3′UTR with a conserved hsa‐miR‐24 seed region (*i.e*. containing G nucleotide) showed a significant (*P* < 0.0005) reduction in luciferase level; on the contrary, luciferase level did not change when the same cells were transfected with a plasmid expressing mutated 3′UTR (containing A nucleotide) (Fig. 1C). These results confirm that *OLR1* is a bona fide miR‐24 target. Moreover, this functional analysis proved that the rs1050286 G‐allele leads to a significant lower luciferase activity (*P* < 0.0005), compared with the A allele, thus demonstrating that *in vitro* interaction among miR‐24 and *OLR1* is impaired by rs1050286 SNP.

**Figure 2:**
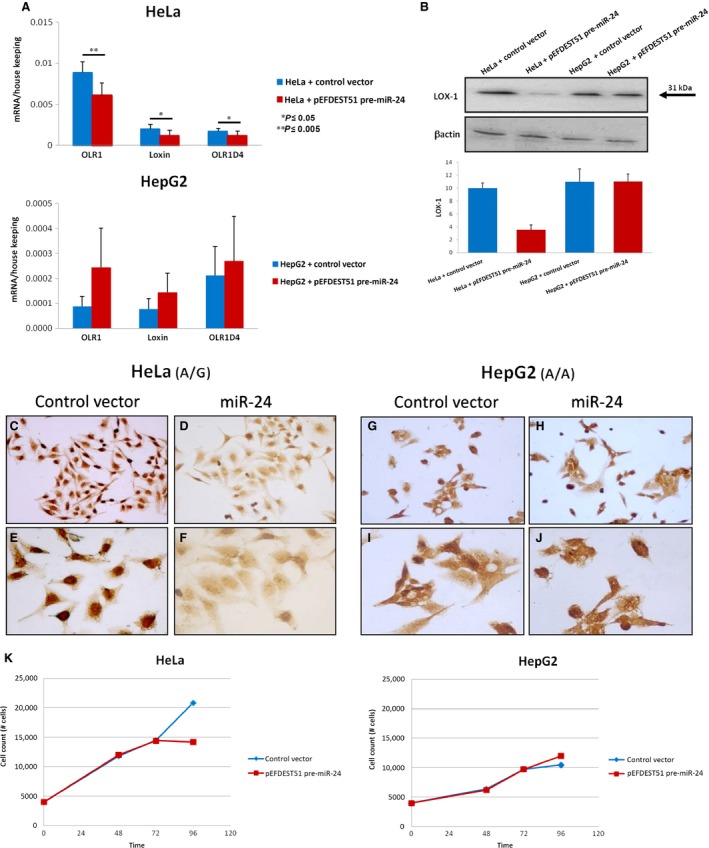
LOX‐1 expression in HeLa and HepG2 cells is modulated by miR‐24 overexpression and rs1050286 SNP. (**A**) qRT‐PCR results. (**B**) Western blot analysis. Bar graphs show the ratio of LOX‐1 to β‐actin. The experiments were repeated three times and the data show the representative results. (**C–K**) Immunocytochemistry results. (**C, E, G, I**) Control plasmid; (**D, F, H, J**) miR‐24 transfected cells. (**C, D, G, H**) 20× magnification; (**E, F, I, J**) 40× magnification. (**K**) HeLa and HepG2 proliferation assay.

To confirm miR‐24/*OLR1* binding, we analysed *OLR1* and its splice isoform expression (mRNA and protein) in two human cell lines with different genotype (A/G *versus* A/A) both at 48 hrs and at 72 hrs after miR‐24 transfection. Although 48 hrs after transfection we did not observe significant changes in *OLR1* mRNA and protein levels (data not shown), 72 hrs post transfection, HeLa cells (A/G) overexpressing miR‐24 showed a significant *OLR1* (and its splice isoforms) mRNA decrease compared to cells transfected with control vector (Fig. 2A). Accordingly, LOX‐1 protein level decreased to about 65% in HeLa overexpressing miR‐24 (Fig. 2B). LOX‐1 down‐regulation by miR‐24 was also evident using Immunocytochemistry experiments (Fig. 2C–F). On the contrary, *OLR1* and its splice isoform expression level (both mRNA and protein) did not change in HepG2 (A/A) after miR‐24 overexpression (Fig. 2A, B, G–J).

Interestingly, 96 hrs after seeding the overexpression of miR‐24 inhibits cell growth in HeLa cells with respect to the same cell line transfected with the empty plasmid, whereas cell growth did not change in HepG2 cells overexpressing miR‐24 compared to the same cells transfected with empty vector (*P* < 0.01, Fig. 2K).

## Discussion

LOX‐1, encoded by the *OLR1* gene, is the major endothelial receptor for ox‐LDL and plays a fundamental role in the pathogenesis of atherosclerosis 1, 2, 4. Moreover, it has been demonstrated that normal LOX‐1 activity is essential for maintaining the structural integrity of tissues; in fact, an increased activity of LOX‐1 is associated with cancer cell invasion 12.

*OLR1* is a spliced gene and its alternative splicing is regulated by six intronic SNPs spanning from intron 4 to 3′UTR; these SNPs are in linkage disequilibrium and related to a higher risk of developing AMI 3, 4. In fact, a specific haplotype (*5′‐CTGGTT‐3′*) correlates with a OLR1/LOXIN ratio 33% higher than those identified in individuals carrying another haplotype (*5′‐GCAAGC‐3*′), and the *5′‐CTGGTT‐3′* haplotype was significantly associated with CAD and myocardial infarction 3, 5.

Based on this and other data, LOX‐1 is generally considered a promising therapeutic target for both atherosclerosis and cancer 13.

Therefore, to identify molecular factors that may regulate the *OLR1* expression, we performed an *in silico* analysis on the coding sequence and 3′UTR of *OLR*1 gene to search putative miRNA binding sites. As functional polymorphisms in 3′UTRs of several genes have been reported to be associated with diseases by affecting gene expression, we searched for SNPs, in the coding sequence and in the UTRs of *OLR1*, which map in the identified miRNA putative binding sites. We found a putative hsa‐miR‐24 binding site that is ‘naturally’ mutated, inside its seed region, by a common SNP (rs1050286) (Fig. 1). In fact, in the European population the frequency of the G allele, that results in a conserved miR‐24 seed region, is 0.483, whereas the A allele has a frequency of 0.517. However, in black/African‐Americans and in East Asians the G allele has a frequency of 0.233 (dbSNP, http://www.ncbi.nlm.nih.gov/).

*In vitro* luciferase assay and overexpression studies demonstrate an interaction among miR‐24 and *OLR1* and also that rs1050286 alters the binding affinity between miR‐24 and its 3′UTR, thus reducing the suppression of *OLR1* expression.

miR‐24 is commonly considered as a multifunctional cardio‐miR that plays good and bad roles in heart; in fact, it protects cardiomyocytes from apoptosis and reduces cardiac fibrosis, but inhibits angiogenesis and deteriorates heart failure 14. Moreover, miR‐24 is up‐regulated in the chronic phase after myocardial infarction and promotes hypertrophic growth of cardiomyocytes in mouse model experiments and the cardiac overexpression of miR‐24 resulted in scar size reduction and heart function improvement 15. Our findings suggest that the rs1050286 SNP in the *OLR1* 3′UTR, by disrupting the regulatory role of miR‐24 on *OLR1* expression, may contribute to the occurrence of atherosclerosis. Moreover, these results highlight the importance of genotype‐dependent differential microRNA regulation in relation to human disease risk.

Finally, these evidence suggest that *OLR1* could be a new therapeutic target for miR‐24 and represent a starting point for the development of possible therapeutic strategies against diseases related to *OLR1* overexpression.

## Conflicts of interest

All authors have no conflicts of interest to disclose relevant to the contents of this paper. The authors confirm that there are no conflicts of interest.

## Author contributions

E.M. and F.A. designed the research study; E.M, B.R and S.P. performed the research; F.F. and H.C.M analysed the data; G.N. and D.C. contributed essential reagents or tools; E.M., G.N. and F.A. wrote the paper.
